# Perspective

**DOI:** 10.1007/s10912-017-9451-7

**Published:** 2017-05-31

**Authors:** Josephine Ensign

**Affiliations:** 1 School of Nursing, University of Washington, Seattle, Seattle, WA USA; 2 Certificate Program in Public Scholarship, University of Washington Simpson Center for the Humanities, Seattle, WA USA; 3 School of Nursing (BS ‘84), Medical College of Virginia/Virginia Commonwealth University, Richmond, VA USA

Now, thirty-two years after graduating from the Medical College of Virginia/Virginia Commonwealth University School of Nursing (BS ‘84) in my hometown of Richmond, I can safely say that the single most important course I took in nursing school was not in nursing. Rather, it was a health humanities and medical ethics course taught in the School of Medicine by a hospital chaplain, Reverend Bob Young. Reverend Bob focused this course on death and dying, and he used a small weekly seminar format with a literary reading and writing group. There were approximately ten students, all first- or second-year medical students, except for me. I was in my first year of undergraduate nursing school and was struggling to avoid both failing and dropping out. I despised nursing school with its antiquated emphasis on rote memorization and rigid hierarchical hospital practice. I vowed never ever to teach or to go near a nursing school again once I graduated.

Now (again), after twenty-one years teaching undergraduate nursing courses at the University of Washington in Seattle, I can safely say that Reverend Bob’s health humanities course is the single-most influential course on my own teaching and healthcare practice. For Reverend Bob’s health humanities course, we completed a final portfolio of poems and prose we had written over the semester as reflection on the course content and on our own personal and professional lives. At twenty-one years of age I wrote some overwrought poems, including one about a baby bird dying in my hands after it had been mauled by my dog. But I also wrote several poems that, if not good by MFA standards, are poems that have stayed with me and helped guide my hands, head, and heart over the many years since I wrote them. Like this one titled “Waiting”:

Sitting on park benches

Wringing their hands

Trying to forget the ill one inside

That hospital there.

The building you just stepped out of

The one you walk by every day

That structure has become a part of the skyline

Seen from the window of a dorm room.

It is a lab, a place to practice

The proper way to give drugs

To make beds

To become a nurse.

But reflected in the eyes of the park bench individuals

The building becomes

One room

One bed

One person

One fear

One hope.

Reverend Young gave me an A-plus for the course. But the grade doesn’t matter as much as the lasting solace his course has given me over the many years of my work as a nurse—and as a nurse educator. Thanks to all of the important hospital chaplains out there—no matter what their faith or spiritual persuasion. And thanks to everyone who works hard to put the human back in health care and in health professions education.

Josephine Ensign, FNP, MPH, DrPH

Associate Professor, University of Washington School of Nursing in Seattle

Affiliate Faculty, University of Washington Simpson Center for the Humanities,

Certificate Program in Public Scholarship Medical College of Virginia/Virginia Commonwealth University School of Nursing (BS ’84)

